# Investigation into the stability and culturability of Chinese enterotypes

**DOI:** 10.1038/s41598-017-08478-w

**Published:** 2017-08-11

**Authors:** Yeshi Yin, Bin Fan, Wei Liu, Rongrong Ren, Huahai Chen, Shaofeng Bai, Liying Zhu, Gang Sun, Yunsheng Yang, Xin Wang

**Affiliations:** 1 State Key Laboratory of Breeding Base for Zhejiang Sustainable Pest, and Key Laboratory for Food Microbial Technology of Zhejiang Province, Institute of Plant Protection and Microbiology, Zhejiang Academy of Agricultural Sciences, 198 Shiqiao Road, Hangzhou, Zhejiang 310021 P. R. China; 2 Department of Gastroenterology and Hepatology, Chinese PLA General Hospital, 28 Fuxing Road, Beijing, 100853 China

## Abstract

Although many gut microbial enterotypes have been reported in Europe, Africa and the U.S., their effects on human health are still not yet clear. Culturing gut microbial enterotypes *in vitro* will be helpful to study their effects and applications. Here, fecal samples from 13 healthy Chinese volunteers were collected and subjected to next-generation sequencing. The results showed that seven of these samples belong to the *Bacteroides* enterotype and another six to the *Prevotella* enterotype. Stability of these Chinese gut microbial enterotypes was also evaluated. Results showed that most of the tested volunteer gut microbiota to be very stable. For one volunteer, the bacterial community returned to the state it was in before intestinal lavage and antibiotics treatment after four months. XP medium was found effective for simulating the *Bacteroides* enterotype independent of the original gut microbial community in an *in vitro* chemostat culture system. Although, the *Prevotella* enterotype was not very well simulated *in vitro*, different culture elements selectively enriched different gut bacteria. Pectin and xylan were found to be related to the enrichment of the genera *Bacteroides*, *Sutterella*, and *Flavonifractor* in this chemostat culture system.

## Introduction

Many studies have shown that intestinal microbiota play important roles in the physiology and pathophysiology of both healthy and diseased hosts. Gut microbes take part in substance metabolism^1^ and regulate immune system development and balance^2^. Fecal bacteria transplantation, though an extreme method, has been used for intervention in some refractory diseases such as *Clostridium difficile* infection^3^, inflamatory bowel disease (IBD) and irritable bowel syndrome (IBS)^4^. In recent years, different gut microbial enterotypes have been identified according to different major bacterial constitutes in African, European, and American individuals^5–7^. Howerer, the physiological effects of these enterotypes and their relationship to disease are still not clear^8^. Although Zhang *et al*. have found enterotypes in obese Chinese children^9^, no *Prevotella* enterotypes have been found in healthy Chinese individuals in any population study^10, 11^. Whether *Prevotella* enterotypes also exist in healthy Chinese people needs further study.

De Filippo *et al*. have reported that diet plays a very important role in shaping gut microbial enterotypes^5^. Wu *et al*. also found long-term dietary patterns to be linked to gut microbial enterotypes^6^. However, the different functions of these enterotypes on host and substance metabolism need further study. Germ-free mice are a tool used for studying the function of gut microbiota^12^. However, Xiao *et al*. reported that only 4.0% of the mouse gut microbial genes were shared (95% identity, 90% coverage) with those of the human gut microbiome^13^. Whether animal experimental results accurately represent what happens in humans has recently aroused wide public concern^14, 15^. Chemostats are *in vitro* systems suitable for the study of interactions and metabolisms of colonic bacteria^16^. With appropriate operational parameters, the major groups of colonic bacteria can be maintained in numbers similar to those observed *in vivo* ^17^. The *in vitro* chemostat system used here may be a suitable alternative tool for the study of enterotype function.

In order to build an *in vitro* model to further evaluate the nutritional requirements for different enterotypes and to generate alternative donor bacterial communities for fecal transplantation, the stability and culturability of Chinese enterotypes were investigated in the current study.

## Results

### Detection of the gut microbial enterotypes in healthy Chinese volunteers

To detect gut microbial enterotypes in healthy Chinese individuals, fecal samples from 13 volunteers were collected in Hangzhou and Beijing. Fecal bacterial genomic DNA was then extracted and sent for 16S rRNA gene V3-V4 region sequencing using the Miseq platform. Basic information of these volunteers and sequencing statistics results are listed in supplementary Tables 1 and 2, respectively. As shown in supplementary Figure 1A, the genus *Bacteroides* was relatively common in four Hangzhou and three Beijing volunteers. These samples were identified as having the *Bacteroides* enterotype. For these volunteers, the percentage of the bacterial population made up of the genus *Bacteroides* ranged from 46.09% to 65.50%. Another five Hangzhou volunteers and one Beijing volunteer had a higher percentage of genus *Prevotella_9*, whose percentage ranged from 48.40% to 87.12%. These were recognized as the *Prevotella* enterotype.

To verify the existence of gut microbial enterotypes in healthy Chinese people, three fecal samples were extracted at another laboratory and sent for sequencing using the 16S rRNA gene V4 region and Hiseq platform. As shown in supplementary Figure 2, the patterns of the bacterial communities were very similar to the Miseq results. The percentage of each genus found between these two methods was used for SPSS software analysis. The similarity coefficients were 0.975, 0.958 and 0.966, respectively (supplementary Figure 2). Bacterial communities of samples N1_T3_Ori and N2_T2_Ori were analyzed further using shotgun metagenome sequencing with 10 Gb sequences obtained for each sample. The 16S rRNA gene reads were aligned to a database for bacterial classification. As shown in supplementary Figure 1B, the genus *Prevotella* was the most common. The relative prevalence of *Prevotella* was 38.54% in NI_T3_Ori and 45.92% in N2_T2_Ori.

### Comparison of Chinese enterotypes to African and European enterotypes

**Figure 1:**
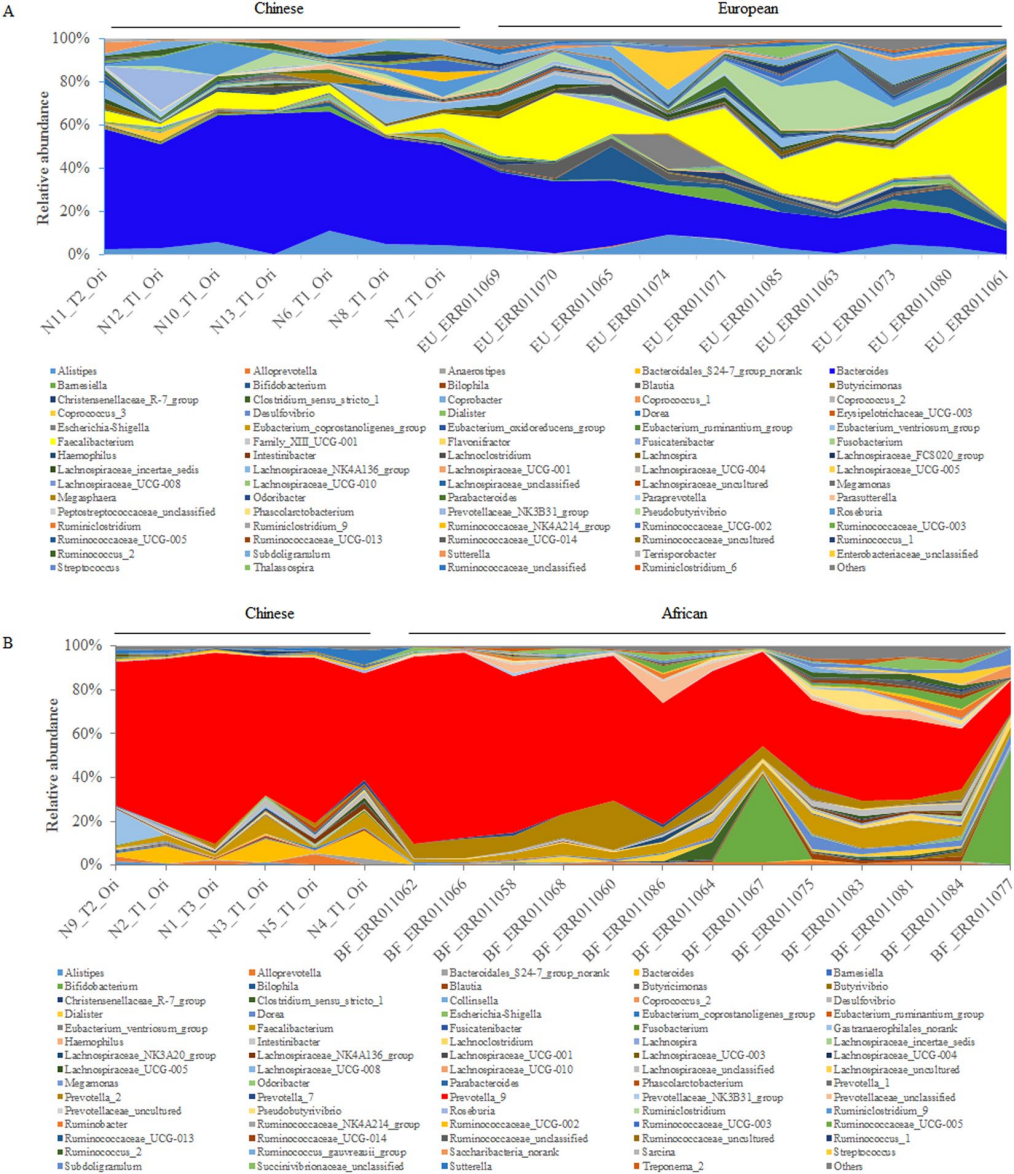
A comparison of the Chinese bacterial community to the European and African communities. Seven *Bacteroides* and six *Prevotella* enterotypes were used in this analysis of Chinese bacterial 16S rRNA gene sequencing data. For European and African bacterial 16 S rRNA gene sequencing data, ten *Bacteroides* and 13 *Prevotella* enterotypes were downloaded from http://www.ebi.ac.uk/ena/data/view/ERP000133. (**A**) Bacterial community of *Bacteroides* enterotype in Chinese and European people. (**B**) Bacterial community of *Prevotella* enterotype in Chinese and African hosts.

Although most countries host *Bacteroides* and *Prevotella* enterotypes, the differences between these enterotypes from different countries are of interesting. African and European enterotype data were downloaded from EMBL and compared to data from the current study. As shown in Fig. 1, although the *Prevotella* enterotype did not show obvious differences between Chinese and African populations (Fig. 1A), Europeans showed a higher prevalence of *Faecalibacterium* and a lower prevalence of *Bacteroides* than the Chinese (Fig. 1B).

### Stability of Chinese enterotypes

**Figure 2:**
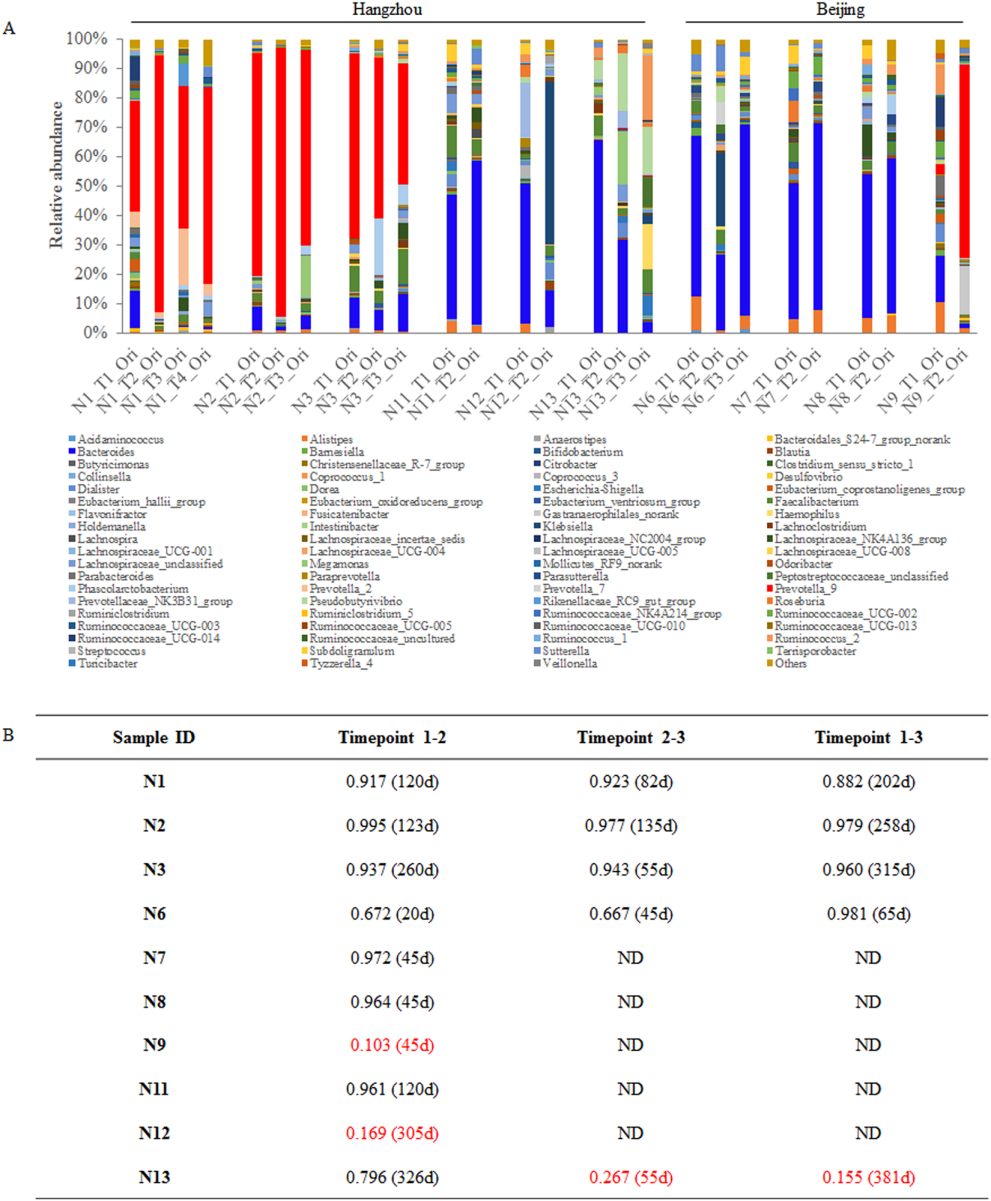
Stability of gut microbial enterotypes in Chinese hosts. Fecal samples were collected from ten healthy volunteers at different points in time. The bacterial community at the genus level is listed in Fig. 3A. The correlation coefficients of gut microbiota at different points in time are listed in Fig. 3B. The number in parentheses represents the number of days between two time points.

In this study, fecal samples were collected from 10 volunteers at different points in time. As shown in supplementary Figure 3, the Shannon index and operational taxonomic unit (OTU) numbers were relatively stable for these samples. The stability of gut microbiota varied by subject. As shown in Fig. 2A, the bacterial community pattern was very similar in the volunteers, except that the *Bacteroides* enterotype of subject N13 changed to *Ruminococcus*, and that of N9 to the *Prevotella* enterotype. For N13, the similarity of gut microbiota was lowest at 15.5% between timepoints 2 and 3. For N9, it was the lowest at 10.3% between timepoints 1 and 2 (Fig. 2B). Although enterotype did not change in N6 and N12, the *Klebsiella* population was visibly increased at one point in time. For N12, the similarity of gut microbiota was lowest at 16.9% between timepoints 1 and 2 (Fig. 2B).

During sample collection, perianal infection was observed in volunteer N1. N1′s *Prevotella* enterotype returned to normal four months later after colon hydrotherapy and antibiotic treatment. As shown in Fig. 2A, the column N1_T4_Ori representsthe gut microbiota of N1 three months before infection, and the column N1_T5_Ori represents that of N1 four months later after disease and treatment. *Prevotella* is the major bacterial genus in these two samples, and the similarity between these two columns was high at 0.927.

### Simulated cultures of the Chinese gut microbial enterotypes

**Figure 3:**
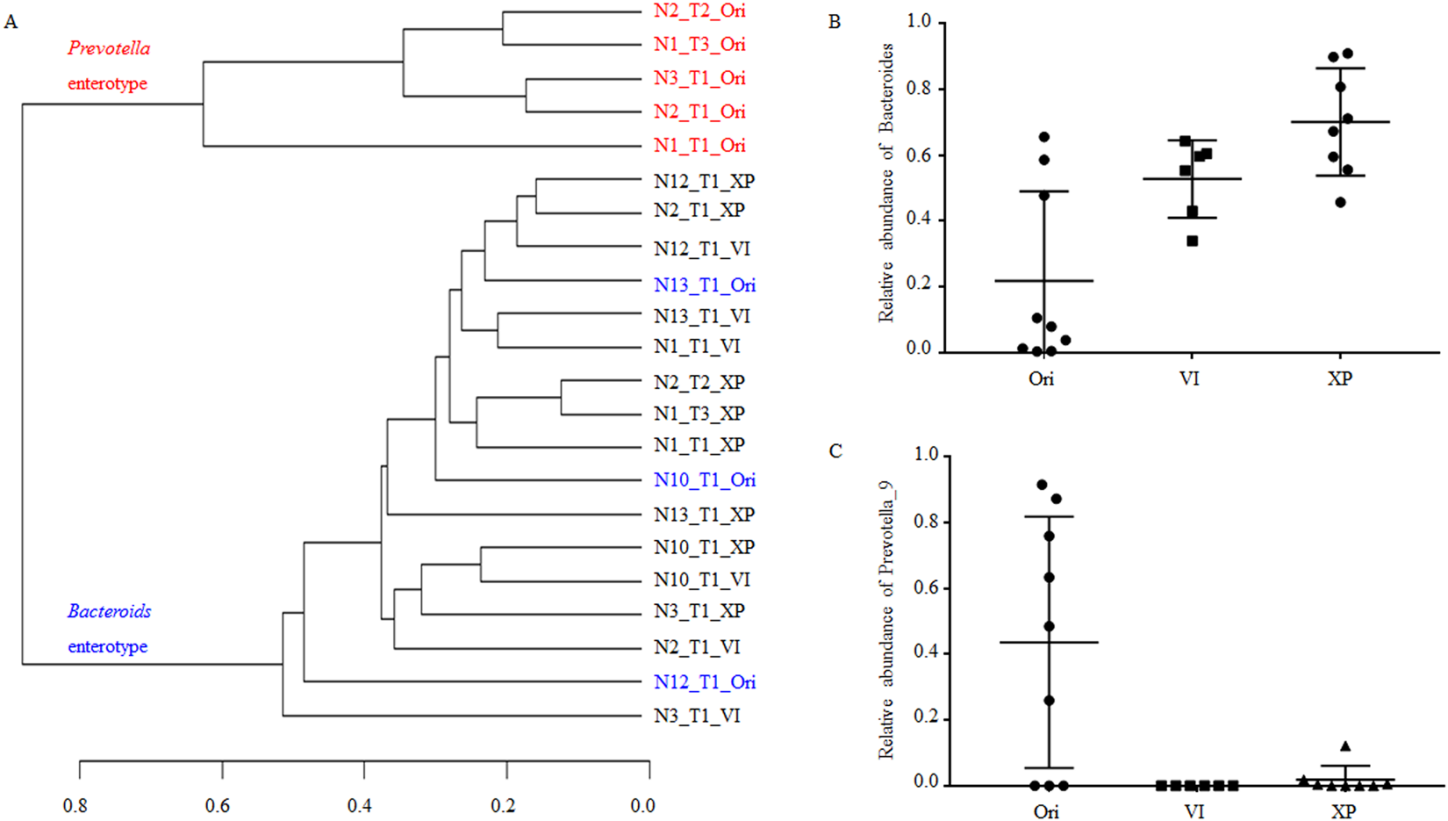
Chemostat simulation of cultures of *Bacteroides* and *Prevotella* enterotypes. Culture media VI, XP, MD1, and MD2 were used to simulate the bacterial communities of *Bacteroides* and *Prevotella* enterotypes *in vitro*. (**A**) Cluster analysis of the relationship among fecal samples and chemostat cultured samples according to the percentage of each genus. (**B**) The percentage of genera *Bacteroides* and *Prevotella* in fecal and chemostat cultured samples.

**Figure 4:**
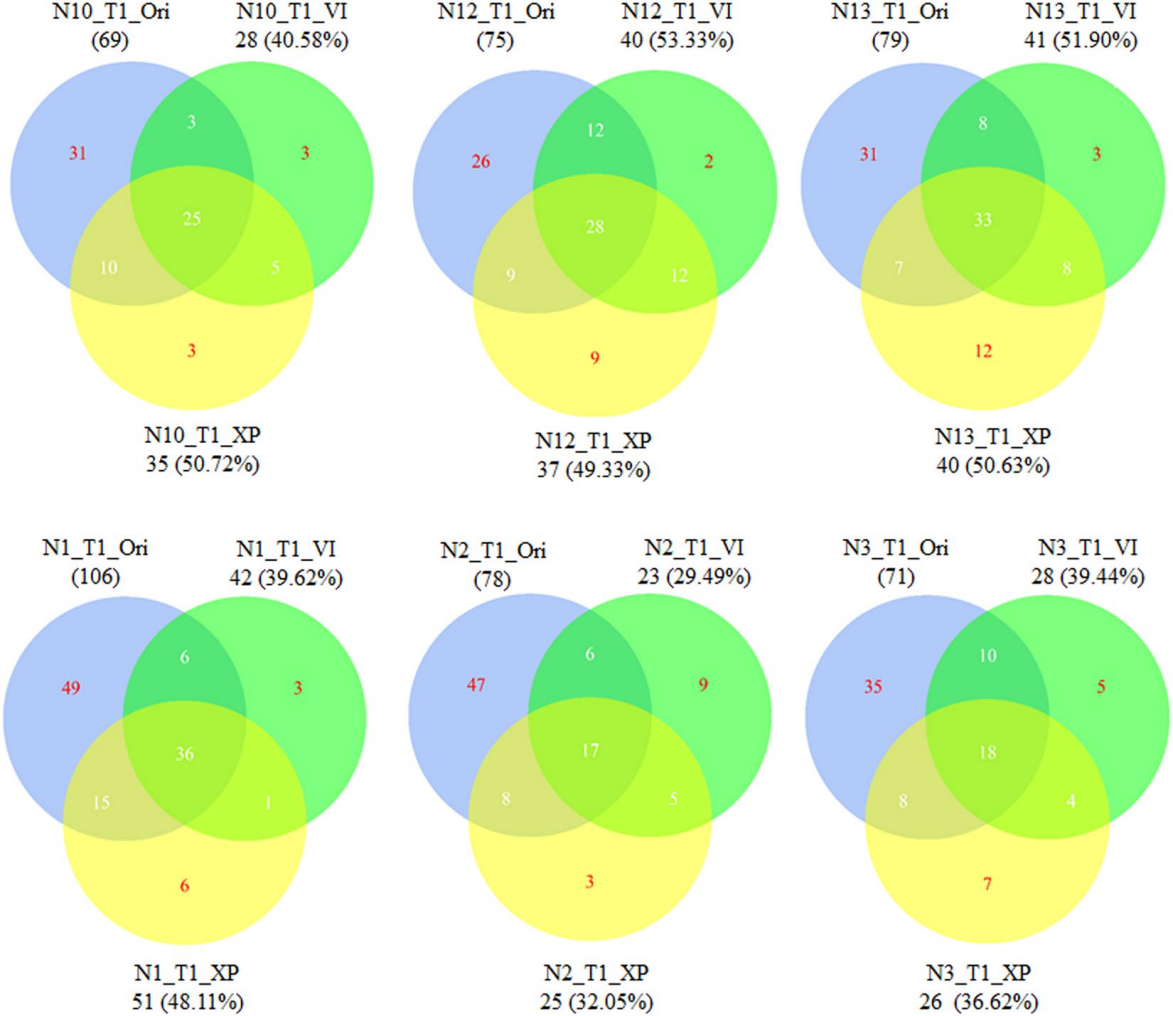
Analyses of the number of genera cultured in chemostats. 16 S rRNA gene high-through put sequencing data was classified using the rdp classifier method and silva123 database. At the genus level, Venn diagrams were then drawn by R package.

VI culture media have been reported to have good effects for simulating culture gut microbiota of the *Bacteroides* enterotype^17^. Wu *et al*. reported that volunteers in whom the *Prevotella* enterotype was predominant favored more sugars^6^. VI and VI with added sugar served as culture media to simulate the gut microbiota of *Bacteroides* and *Prevotella* enterotypes. After ten days of continuous culture, the bacterial communities in chemostats became stable as indicated by PCR-DGGE results (supplementary Figure 4). Fermentation products collected on day 13 were then collected for 16S *rRNA* gene sequencing. As shown in supplementary Figure 5A and Fig. 3, both VI and XP can simulate the gut microbiota of the *Bacteroides* enterotype, with the similarity between the original fecal bacterial community and the chemostat culture products being higher than 80% (Table 1). 40–50% of original fecal genus remained alive in the chemostat after 13 days of continuous culture (Fig. 4). However, the simulated efficiency of VI and XP media for the *Prevotella* enterotype was not as strong as expected (supplementary Figure 5B, Fig. 3A and C). Although 12.11% of *Prevotella* genus was detected in N1_T1_XP (supplementary Figure 5B), the majority of *Prevotella* bacteria did not grow very well in the chemostat (Fig. 3C and supplementary Figure 5B). The similarity of bacterial communities in the original fecal sample and chemostat culture product was below 5% (Table 1), and only 29.49–61.84% of fecal genera could survive in the chemostat (Fig. 4). However, bacterial communities in all VI and XP fermentation products were very similar to the *Bacteroides* enterotype (Fig. 3A). The similarity coefficient was over 85%, even from inocula that originated from the *Prevotella* enterotype (Table 1).

**Table 1:** Similarity of gut microbiota between original human fecal samples and fermentation products.

Sample ID	N10_T1_Ori	N12_T1_Ori	N13_T1_Ori
N10_T1_Ori	1.000	0.970	0.958
N10_T1_VI	0.845	0.876	0.897
N10_T1_XP	0.932	0.964	0.974
N12_T1_Ori	0.970	1.000	0.980
N12_T1_VI	0.850	0.877	0.884
N12_T1_XP	0.953	0.981	0.986
N13_T1_Ori	0.958	0.980	1.000
N13_T1_VI	0.948	0.972	0.976
N13_T1_XP	0.844	0.865	0.863
N1_T1_Ori	0.077	0.081	0.083
N1_T1_VI	0.940	0.973	0.967
N1_T1_XP	0.937	0.964	0.967
N2_T1_Ori	0.077	0.076	0.082
N2_T1_VI	0.915	0.948	0.951
N2_T1_XP	0.955	0.984	0.988
N3_T1_Ori	0.144	0.135	0.148
N3_T1_VI	0.652	0.645	0.603
N3_T1_XP	0.925	0.945	0.932

Ori, original fecal sample; VI and XP, fermentation products using VI and XP culture media, respectively.

**Figure 5:**
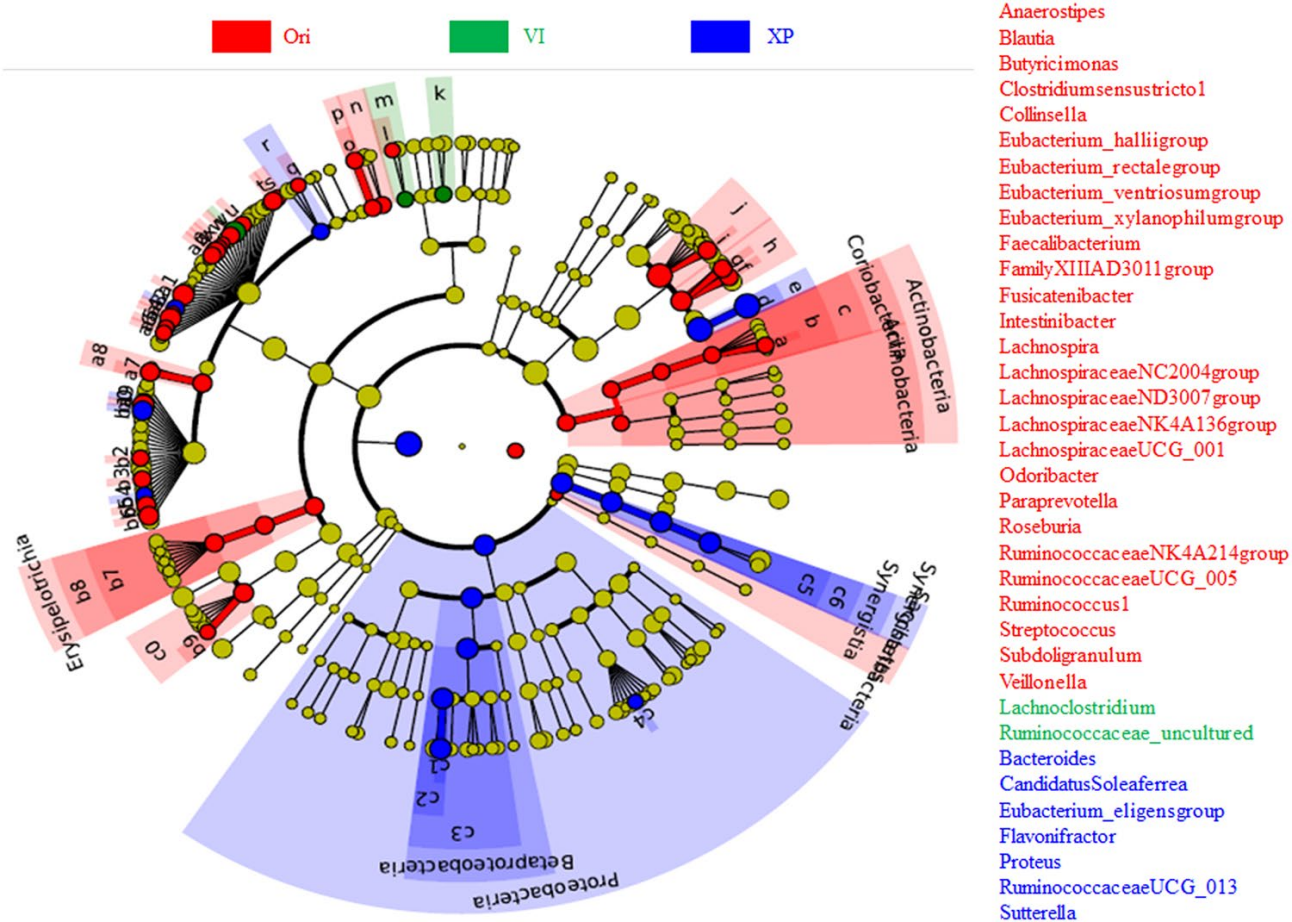
Analysis of different abundant bacterial taxa using LefSe. The bacterial percentage of original fecal samples and fermentation samples was used for LefSe analysis. The *p* value < 0.05 was identified as significantly different among these groups. Significantly enriched genera are listed on the right side.

Though *Bacteroides* was the predominant genus cultured in both VI and XP media, 27 genera were significantly higher in the original fecal samples (Fig. 5). And different culture media selectively enriched different bacteria. As shown in Fig. 5, only the genera *Lachnoclostridium* and uncultured *Ruminococcaceae* were enriched after culture *in vitro* for six volunteers using VI media. The principal carbon source of the medium is soluble starch. For XP media, partially soluble starch is replaced with pectin and xylan. The genera *Eubacterium_eligensgroup, Bacteroides, Candidatus Soleaferrea, Flavonifractor, Proteus, RuminococcaceaeUCG_013* and *Sutterella* were selectively increased.

To improve the simulation of *Prevotella* growth, glucose and sucrose were added to the medium to serve as a carbon sources (MD1 and MD2) and beef extract as a nitrogen source (VL). Vitamin K and aspartic acid were also added into the chemostat (VI1 and VI2). Although *Prevotella* still did not grow very well *in vitro* (supplementary Figure 5B), and the similarity was still very low between the original fecal samples and chemostat-cultured products (supplementary Figure 5B), more genera (41.79–61.84%) were cultured in the chemostat (supplementary Figure 5C).

### Detection of SCFA production

**Figure 6:**
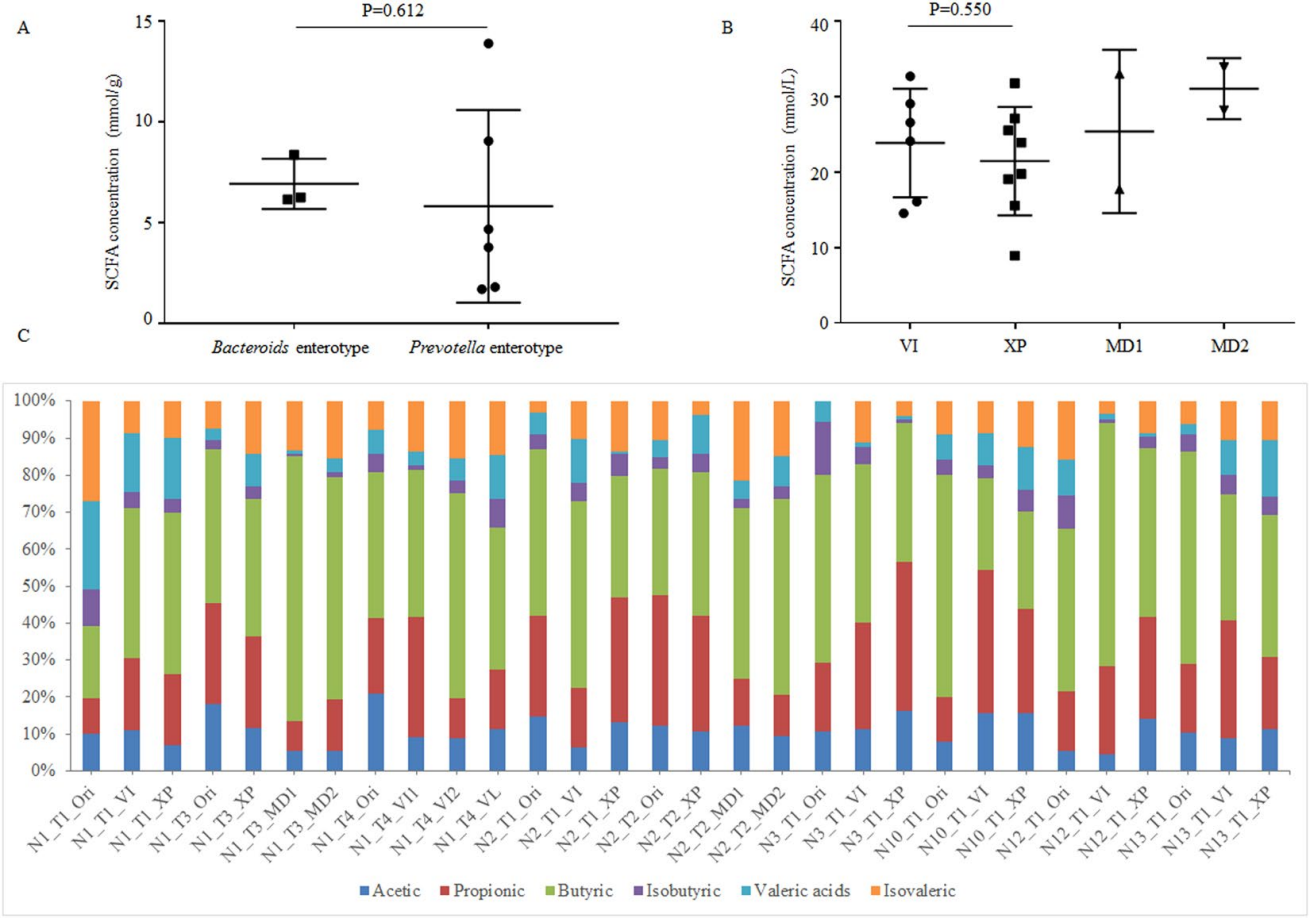
Total SCFA concentrations and relative percentages of SCFAs in fecal and fermentation samples. Acetic, propionic, isobutyric, butyric, isovaleric, and valeric acids were detected in the present study, and the total SCFA represents all SCFAs. The concentrations of SCFAs in the original fecal samples and batch chemostat were assessed using gas chromatography (GC). (**A**) Total concentration of SCFAs in original fecal samples (mmol/g). (**B**) Total concentration of SCFAs in fermentation samples (mmol/L). (**C**) The relative percentage of these SCFAs in each sample.

Before inoculation, short-chain fatty acid (SCFA) concentrations of the original fecal samples were measured. As illustrated in Fig. 6A, the differences in SCFA concentrations between *Bacteroides* and *Prevotella* enterotypes were not significant. And the relative percentage patterns of SCFAs between *Bacteroides* enterotype (volunteers N1_Ori, N2_Ori, and N3_Ori) and *Prevotella* enterotype (volunteers N10_Ori, N12_Ori, and N13_Ori) were similar (Fig. 6C). For fermentation samples, the SCFA patterns were similar among the different culture media (Fig. 6C), and the production of SCFAs using different culture media was not significantly different (Fig. 6B).

## Discussion

In this study, we reported the existence of the *Prevotella* enterotype in healthy Chinese individuals. Five volunteers from Hangzhou and one volunteer from Beijing were found have a higher percentage of *Prevotella* in fecal samples (supplementary Figure 1). Different sequence platforms and shotgun metagenomic analysis further verified the existence of the *Prevotella* enterotype in healthy Chinese individuals (supplementary Figures 1B and 2). Combined with the results from Zhang *et al*.^9^, this is consistent with a previous study that found *Bacteroides* and *Prevotella* enterotypes to be present in both healthy and diseased individuals^8^.

The stability of gut microbiota is the theoretical basis for targeted disease treatment. Although some studies have shown that the gut bacterial community becomes stable after the host reaches adulthood^18, 19^, Knights *et al*. suggested that the enterotype distribution is continuous^8^. The stability of Chinese gut microbial enterotypes needs to be studied further. In the present work, the gut microbiota of six of the ten volunteers was very stable. Participant N1′s intestinal bacterial community returned to its pre-disease state four months after intestinal lavage and antibiotic treatment (Fig. 2). However, Browne *et al*. have found that antibiotics treatment, especially in children, changes the gut bacterial community long-term^20^. Keeping the gut microbiota stable is possible in adults, even those who have experienced short-term, severe disorders, if they maintain consistent living and eating habits.

Fecal bacteria transplantation is one of the more extreme methods of intervention for intestinal dysbiosis. In 2013, van Nood *et al*. reported the treatment effects of fecal bacteria transplantation on *Clostridium difficile* infection^3^. Other diseases treated using fecal bacteria transplantation have included IBD and IBS^4^. However, most of the donors are patients’ relatives, and samples were collected for fresh use. Only a small number of fecal samples can be collected from one person at a time, so the microbial community for donors is different every time and the risk also varies due to differences in the bacteria. This has hindered the extensive commercial application of fecal bacteria transplantation. An *in vitro* chemostat system maybe an alternative method to produce fecal bacteria transplantation products for human use. In this study, cultured gut microbes were simulated *in vitro* using a continuous chemostat system, and the similarity between fermentation products and original fecal samples was higher than 80% for the *Bacteroides* enterotype. The degree of simulation was not affected by the original enterotype (Table 1). The *Bacteroides* enterotype was identified after culture *in vitro* using *Bacteroides* and *Prevotella* enterotypes as inocula, which may be convenient for donor selection. At the genus level, bacterial communities found in Chinese hosts were very similar to those of African and European hosts (Fig. 1). This provides a basis for future use of the fermentation products as fecal bacteria transplantation donors.

Diet is one of the most important factors affecting the gut microbial community^21, 22^. De Filippo *et al*. inferred that diet may be the major reason for different enterotypes^5^. Kovatcheva-Datchary *et al*. reported the prevalence of *Prevotella* to be higher after the addition of more fiber to the diet^23^. A diet questionnaire filled out by American participants also showed that *Prevotella* volunteers favored more sugars, especially monosaccharides^6^. Unfortunately, it was not possible to simulate the bacterial community of the *Prevotella* enterotype (Fig. 3 and supplementary Figure 5), even with added glucose. We also tried to modulate nitrogen, amino acids, and vitamines to improve the simulation. However, the genus *Prevotella* still did not grow well in chemostats. We therefore speculate that *Prevotella* growth require nutrition factors originating from the host. Wu *et al*. reported that host factors may have a strong effect on gut microbiota^24^. Gut-chip-like fermentation equipment has been used to culture host cells and gut bacteria simultaneously^25–27^. This may improve the simulation of *Prevotella* culture *in vitro*.

Although the relationship between enterotypes and different diseases is still not clear^8^, diet may be the major reason for different enterotypes^5, 6^. SCFAs, as the key gut bacterial metabolites produced by fermenting dietary fiber, may be the link between diet, enterotype, and host physiology^28^. Chen *et al*. recently reported that the *Prevotella* enterotype can ferment fructooligosaccharides, sorghum, and corn arabinoxylans and significantly promote higher propionate and total SCFA production compared to the *Bacteroides* enterotype^29^. In this study, we found that the total concentration and the relative percentage of SCFAs was similar between *Prevotella* and *Bacteroides* enterotypes (Fig. 6). And the total concentrations and relative percentages of SCFAs in fermentation products using different culture media were also similar (Fig. 6). This discrepancy may be due to the use of only one *Prevotella* and one *Bacteroides* enterotype sample in the Chen *et al*. study. The use of different molecular weight carbohydrates (polysaccharides *vs* oligosaccharides and monosaccharides) may also affect SCFA production. The nutritional requirements and metabolic properties of different enterotypes still require further study.

Results showed that different sources of carbon selectively enriched different gut bacteria (Fig. 5), which will be helpful for gut bacterial isolation. Lau *et al*. reported that some bacterial OTU cannot be detected using next-generation sequencing due to the nature of PCR and low abundance^30^. Selection isolation may be an alternative method for gut bacterial detection. The current results also showed that many genera cannot be detected in their original samples, but were rather detected after culture *in vitro* (Fig. 4 and supplementary Figure 5C). Different culture media selectively enriched different bacteria. For samples from six of the volunteers, only the genera *Lachnoclostridium* and uncultured *Ruminococcaceae* were enriched in VI media. *Bacteroides*, *Sutterella* and *Flavonifractor* were enriched using XP culture media. Enrichment of the genera *Sutterella* and *Flavonifractor* may have been related to the replacement of 4 g of soluble starch with 2 g pectin and 2 g xylan. In this way, selective enrichment cultures of gut microbiota may be helpful to isolate newly discovered bacteria and bacteria with low abundance.

In summary, our results showed *Bacteroides* and *Prevotella* enterotypes to be present in healthy Chinese adults. Gut microbial communities remained stable for most of the people tested, even those who experienced short-term but severe disorders. The bacterial community of the *Bacteroides* enterotype was simulated well *in vitro*, independent of the original enterotypes.

## Materials and Methods

### Origin of human fecal samples

Fecal samples of 9 healthy human volunteers (6 males and 3 females), ranging in age from 25 to 38 years old, were collected from Hangzhou. Fecal samples of 4 healthy human volunteers (1 male and 3 females), ranging in age from 10 to 20 years old, were collected from Beijing. For 10 volunteers, fecal samples were collected more than once. Exclusion criteria included recent antibiotic treatment, frequent gastrointestinal disorders, and metabolic disease. This study was carried out according to the Helsinki Declaration and informed written consent was obtained from all human subjects. Details regarding age, gender, and sample collection times are listed in supplementary Table 1. About 2 g of fresh fecal samples were collected immediately after defecation, placed in an ice box, and stored at −80 °C for further analysis. The study was approved by the Ethics Committee of the Zhejiang Academy of Agricultural Sciences and PLA General Hospital.

### DNA extraction

Bacterial genomic DNA was extracted using a QIAamp DNA Stool Mini Kit according to the manufacturer’s instructions (Qiagen, German). The concentration of extracted DNA was determined using a NanoDrop ND-2000 (NanoDrop Technologies, USA), and its integrity and size were confirmed by agar gel electrophoresis (1.0%). To determine the quality of the DNA, primers of 341F (5′-ATT ACC GCG GCT GCT GG-3′) and 534R (5′-CCT ACG GGA GGC AGC AG-3′) were used to amplify the V3 region of the bacterial 16S rRNA gene.

### 16S rRNA gene sequencing and analysis

According to previous work, bacterial 16S rRNA genes were amplified from extracted DNA using barcoded primers 338F (5′-ACT CCT ACG GGA GGC AGC A-3′) with 806R (5′-GGA CTA CHV GGG TWT CTA AT-3′)^31^, 515F(5′-GTG CCA GCM GCC GCG GTA A-3′) with 806R (5′-GGA CTA CHV GGG TWT CTA AT-3′)^32^ or 515F with 907R (5′-CCG TCA ATT CMT TTR AGT TT-3′)^33^. Most next-generation sequencing was performed using an Illumina MiSeq. 300PE, which was conducted by Majorbio Bio-Pharm Technology Co., Ltd., Shanghai, China. Comparisons of the taxonomic differences in sequences were generated by different labs, the bacterial genomic DNA of three fecal samples was extracted at Beijing and Hangzhou by different students, and were sent for sequencing on a Hiseq platform at Novogene (Beijing, China) and MiSeq platform at Majorbio (Shanghai, China), individually. Details of the primers and platforms used in this study are given in supplementary Table 2.

Next-generation sequencing reads generated in this study and data downloaded from the European Nucleotide Archive (ERP000133) were identified by barcodes using QIIME pipeline^34^. Clean, high-quality sequences were then used for downstream analysis. A 97% similarity cutoff was used to define OTU by using Mothur^35^. One sequence from each OTU was selected and considered representative. These sequences were classified using the rdp classifier method^36, 37^ and silva123 database (https://www.arb-silva.de/documentation/release-123/). Good’s coverage, alpha diversities including Simpson and Shannon indexes, and richness (observed number of OTUs) were calculated using Mothur.

### Metagenomic sequencing and analysis

To further verify the existence of the *Prevotella* enterotype in Chinese hosts, one male and one female fecal sample were randomly selected for shotgun sequencing. Bacterial genomic DNA was extracted using a QIAamp DNA Stool Mini Kit, and 300 bps libraries were constructed using Covaris M220, a TruSeq™ DNA Sample Prep Kit and a cBot Truseq PE Cluster Kit v3-cBot-HS. A Truseq SBS Kit v3-HS (200 cycles) was then used for sequencing on an Illuminal Hiseq platform. The bacterial 16S rRNA gene sequences were then picked out for taxa analysis.

### Single-stage chemostat fermentation

A parallel chemostat system containing two single-stage chemostats systems (330 ml working volume) was set up as described previously by Yin *et al*.^38^. The pH (6.2) was automatically controlled using a pH controller, and the temperature (37 °C) was maintained using a circulating water bath. The systems were kept anaerobic by continuous sparging with O_2_-free N_2_ and operated at a dilution rate of 0.04 ml/h.

Based on the enterotype data, some volunteers’ intermediate partial fecal samples were collected for homogenizing in stomacher bags with 0.1 M anaerobic PBS to make 10% (wt/vol) slurries. Large particles of food residue were removed by passing the mixture twice through a 0.4 mm sieve. Then 30 ml of human fecal slurry was inoculated into the parallel chemostat system containing different culture media (Table 2). After overnight equilibration, fresh medium was added to the system using a peristaltic pump. The system was equilibrated for 7 days before samples (15 ml) were collected for DNA extraction and sequencing. In this study, veal infusion broth (VI medium) containing mainly polymerized carbon and protein^39, 40^, and Viande Levure (VL) medium, containing mainly hydrolyzed nitrogen and simple sugars^41^, were used as the basal culture media. Simple sugar^24^, vitamins^42^ and amino acids^43^ were added given that some studies have found that these components maybe related to *Prevotella* growth.

**Table 2:** Components of the culture media used in this study.

	VI	XP	MD-1	MD-2	VI-1	VI-2	VL
Carbon source (g/L)	Starch 8	Starch 4; Pectin 2; Xylan 2	Glucose 12	Glucose 6; Sucrose 6	Glucose 8	Glucose 8	Glucose 2.5
Nitrogen source (g/L)	Tryptone 3; Peptone 3; Yeast extract 4.5						Tryptose 10; Beef extract 2.4; Yeast extract 5
Trace elements (mg/L)	MgSO4·7H2O 6; CaCl2·2H2O 0.2; MnCl2·4H2O 0.64; FeSO4·7H2O 0.2; CoSO4·7H2O 0.36; ZnSO4·7H2O 0.36; CuSO4·5H2O 0.02; NiCl2·6H2O 0.184.						
Vitamin (μg/ml)					Vitamin K 0.5		
Amino acid					Aspartic acid 0.5%		
Others (g/L)	Mucin 0.5; Bile salts 3# 0.4; L-cysteine hydrochloride 0.8; Hemin 0.05; Tween 80 1 ml; NaCl 4.5; KCl 2.5; MgCl2·6H2O 4.5; CaCl2·6H2O 0.2; KH2PO4 0.4;						L-cysteine hydrochloride 0.6; NaCl 5

### PCR-DGGE analysis

To evaluate the stability of the chemostat system *in vitro*, the microbial communities were analyzed using PCR-DGGE. The V3 region of the 16S rRNA gene (positions 341 to 534 of the *Escherichia coli* gene) was analyzed using PCR-denaturing gradient gel electrophoresis (DGGE) as described previously^38^. DGGE was performed using a DCode universal mutation detection system (Bio-Rad, Hercules, CA, USA) in an 8% (wt/vol) polyacrylamide gel containing a linear 30%-to-60% denaturant gradient with a constant voltage of 200 V at 60 °C for 4 h. The gels were then visualized by staining with SYBR green I nucleic acid (Sigma, St. Louis, MO, USA) for 45 min and washed twice with deionized water. The DGGE profiles were analyzed for similarity using Quantity One software (version 4.6.1; Bio-Rad, USA).

### Short chain fatty acid (SCFA) analysis

SCFA production was determined by using GC as previously described^44^. Briefly, 1 mL of the fermentation products or 10% (wt/vol) fecal slurries were mixed with 0.2 mL of 25% (w/v) metaphosphoric acid. The samples were subsequently centrifuged (14, 000 g for 20 min), and the supernatant was used for SCFA determination (Shimadzu, GC-2010 Plus, Japan). An InertCap FFAP column (0.25 mm × 30 m × 0.25 μm) was used in this study. Peaks were integrated using GC Solution software, and SCFA content was quantified by using the single-point internal standard method. Peak identity and internal response factors were determined using a 20-mM calibration cocktail that included acetic, propionic, isobutyric, butyric, isovaleric, and valeric acids.

### Statistical analysis

To compare Illumina-based high-throughput sequencing data, Pearson correlation coefficients were analyzed using SPSS software (version 20.0; SPSS Inc., USA). The correlation coefficients among samples were calculated based on the percentage of each bacterial classification unit at the genus level. Plot cladograms and significantly different bacterial taxa were analyzed using LefSE Software (https://bitbucket.org/biobakery/biobakery/wiki/lefse). The SCFAs of each sample were measured in triplicate. The differences between means were assessed by the SPSS software. P < 0.05 was considered statistically significant.

### Data availability

All high-throughput sequencing data in the present study have been deposited in the sequence read archive (SRA) of the NCBI database under number SRP082347. The shotgun sequencing data have been deposited in the NCBI database under numbers SRR4254099, SRR4254100, SRX2174002, and SRX2174003.

## Electronic supplementary material

Supplementary Information
