# Supplementary material for: Investigation into the stability and culturability of Chinese enterotypes
Source: Sci Rep. 2017 Aug 11;7:7947. doi: 10.1038/s41598-017-08478-w (PMC5554170; doi:10.1038/s41598-017-08478-w)
Supplement: Supplementary file 1 — Supplementary Information [file 41598_2017_8478_MOESM1_ESM.pdf]

Supplementary materials for

Investigation into the stability and culturability of Chinese enterotypes

Yeshi Yin[1, #], Bin Fan[2, #], Wei Liu[1], Rongrong Ren[2], Huahai Chen[1], Shaofeng Bai[1], Liying Zhu[1], Gang Sun[2], Yunsheng Yang[2, *], Xin Wang[1, *]

[1]State Key Laboratory of Breeding Base for Zhejiang Sustainable Pest, and Key Laboratory for Food Microbial Technology of Zhejiang Province, Institute of Plant Protection and Microbiology, Zhejiang Academy of Agricultural Sciences, 198 Shiqiao Road, Hangzhou, Zhejiang 310021, P. R. China.

[2]Department of Gastroenterology and Hepatology, Chinese PLA General Hospital, 28 Fuxing Road, Beijing 100853, China.

[#]These authors contributed equally to this work.

[*]Correspond authors, xxww101@sina.com and sunny301ddc@126.com.

Supplementary Figure 1. Detection of the gut microbial enterotypes in China. Fecal samples were collected from nine healthy volunteers from Hangzhou and four from Beijing. Bacterial genomic DNA was extracted. All samples were sent for high-throughput 16S rRNA gene sequencing, and two genomic DNA samples were sent for shotgun metagenome sequencing. Bacterial communities assessed at the genus level using 16S rRNA gene sequencing are listed in A. Bacterial communities assessed at the genus level using shotgun metagenome sequencing are listed in B.

A

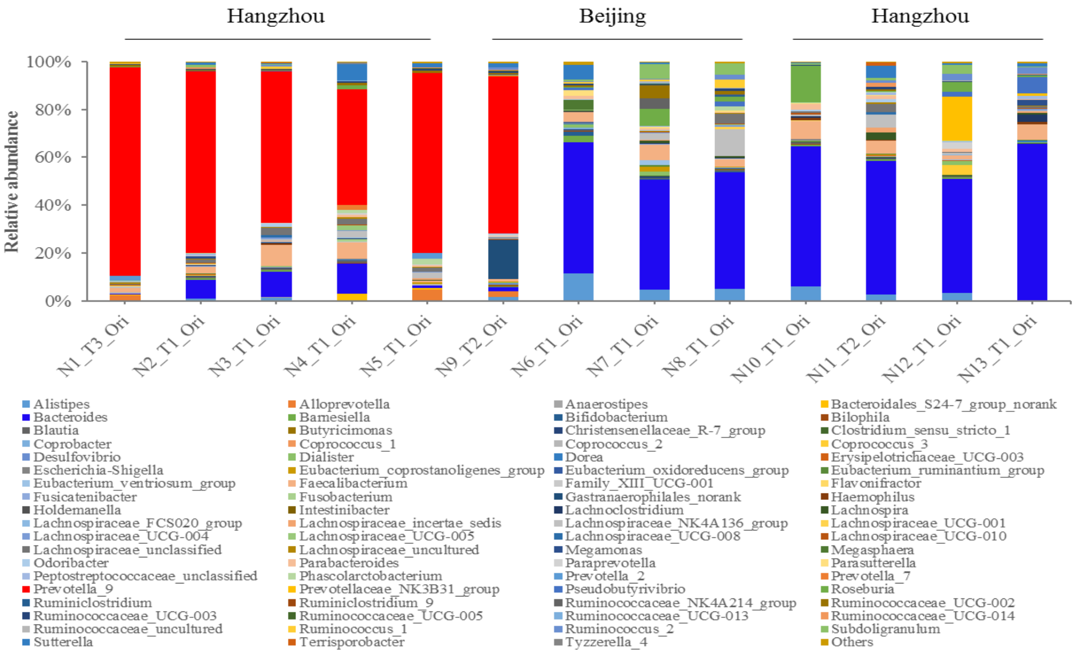

B

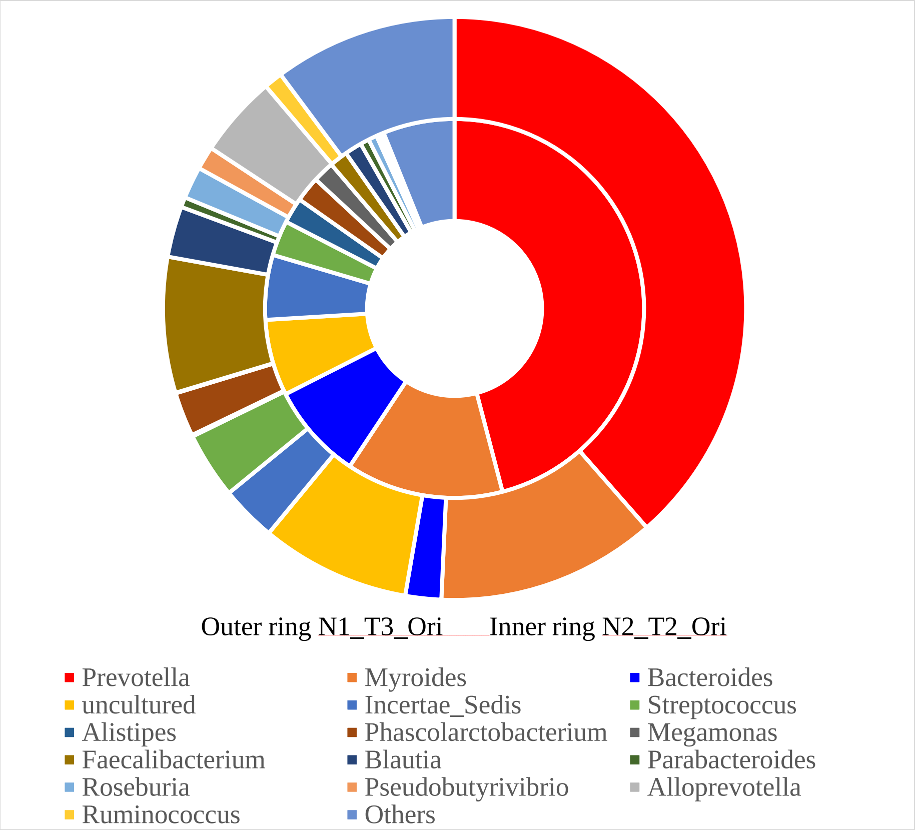
Outer ring N1_T3_Ori
Inner ring N2_T2_Ori
Prevotella
Myroides
Bacteroides
uncultured
Incertae_Sedis
Streptococcus
Alistipes
Phascolarctobacterium
Megamonas
Faecalibacterium
Blautia
Parabacteroides
Roseburia
Pseudobutyrivibrio
Alloprevotella
Ruminococcus
Others

Supplementary Figure 2. Different sequencing platforms and the bacterial community.

Fecal samples collected from volunteers N7, N8, and N9 were separated into two parts. One was sent for bacterial genomic DNA extraction from a Hangzhou laboratory, and the other one was extracted at a Beijing laboratory. DNA extracted from Hangzhou was sent to sequence in the 16S V3-V4 region using Miseq, and results were marked as N7_T2_Miseq, N8_T2_Miseq and N9_T2_Miseq. DNA extracted from Beijing was sent to sequence the 16S V4 region using the Hiseq platform, and results were marked as N7_T2_Hiseq, N8_T2_Hiseq and N9_T2_Hiseq. The similarity of bacterial communities between two the sequencing platforms was calculated using SPSS software. The similarity coefficients are listed at the top of the figure.

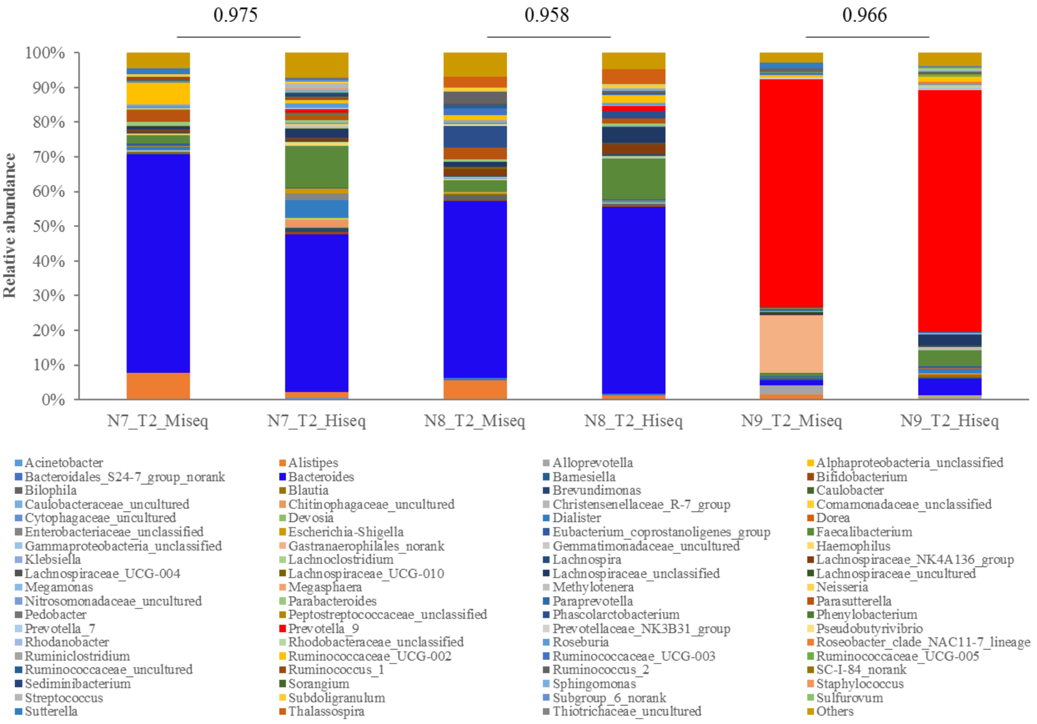

Supplementary Figure 3. Stability of Shannon index and OTU number in Chinese samples.

Fecal samples were collected from ten healthy volunteers at different points in time. A, Shannon index for these volunteers at different timepoints. B, OTU numbers of these volunteers at different timepoints.

A

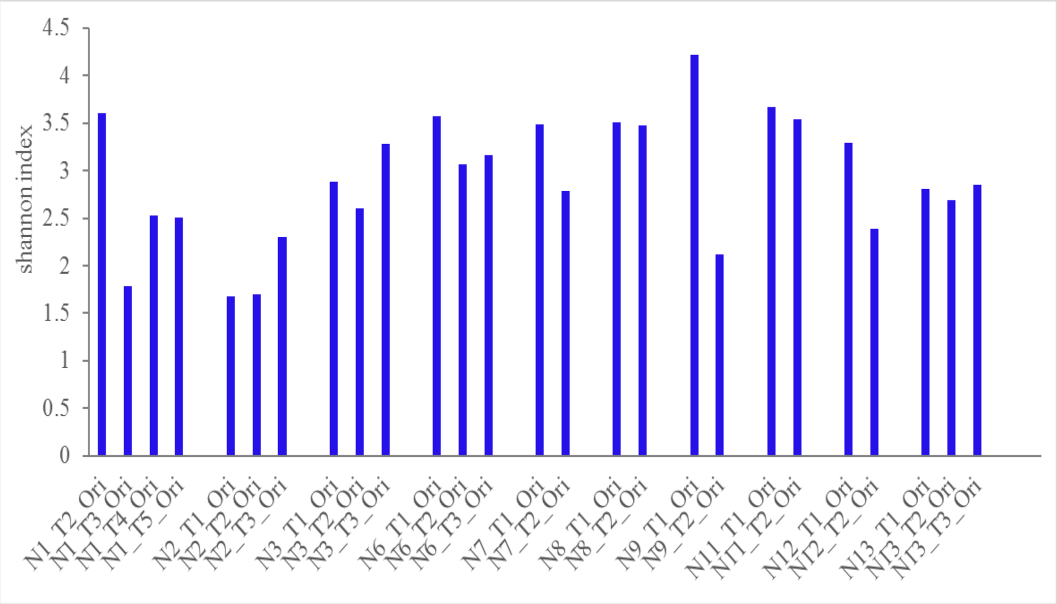

B

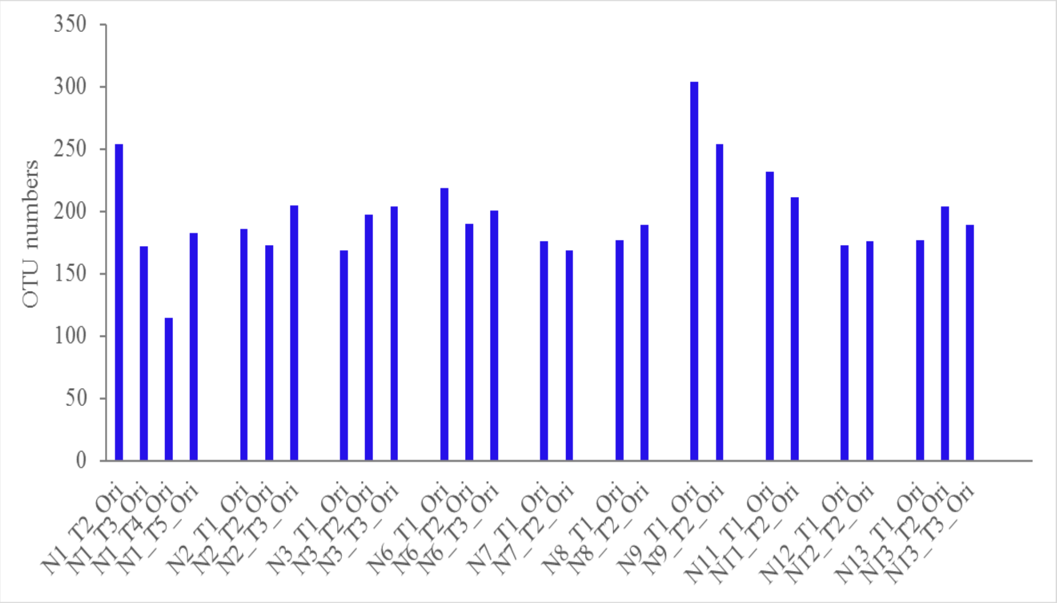

Supplementary Figure 4. PCR-DGGE analysis of the stability of the chemostat system.

Fecal samples and fermentation products were collected for extraction of bacterial genomic DNA. PCR-DGGE was then used to analyze the bacterial community. O, original fecal samples; VI and XP, fermentation products cultured using VI and XP media, respectively; M, the DNA marker; 8-14, days after fermentation.

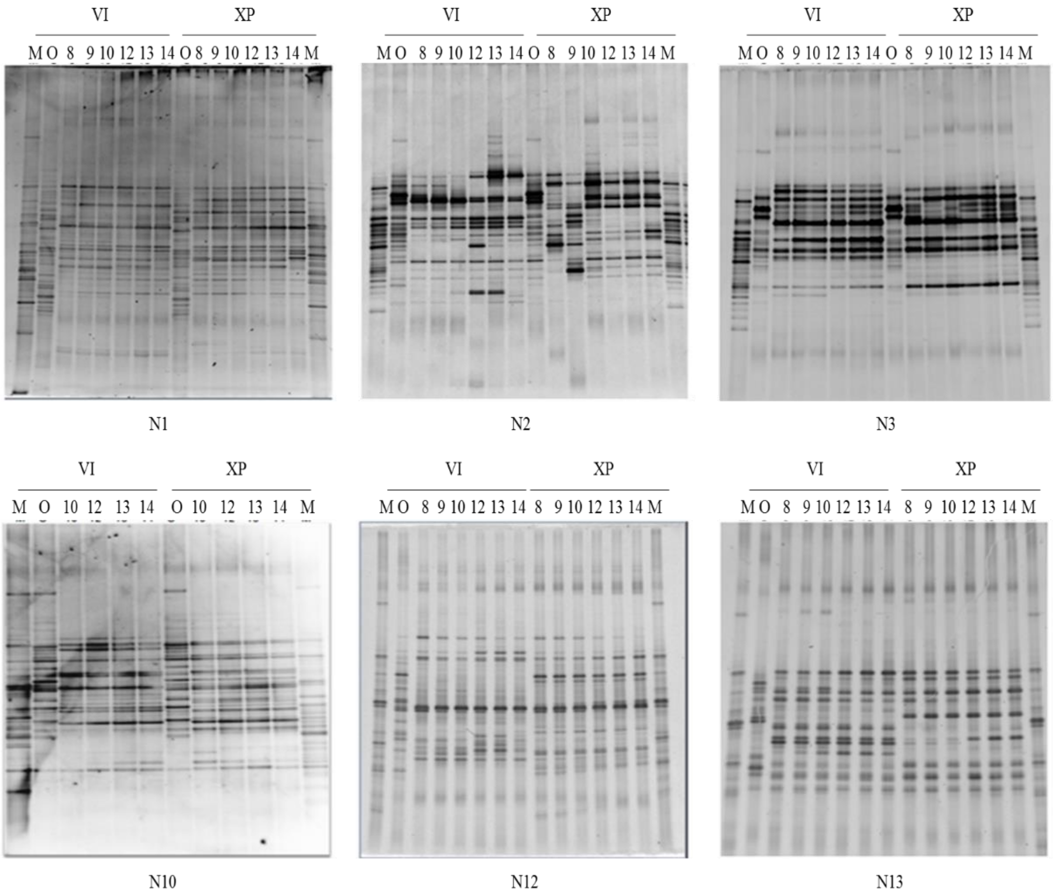

Supplementary Figure 5. Bacterial communities of fecal samples before and after chemostat culture.

Culture media VI, XP, MD1, MD2, VI1 and VI2 were used to simulate the bacterial communities of *Bacteroides* and *Prevotella* enterotypes *in vitro*. A, simulated culture bacteria of the *Bacteroides* enterotype. B, simulated culture bacteria of the *Prevotella* enterotype. The similarities of bacterial communities between original fecal samples and fermentation products were calculated using SPSS software. The similarity coefficients are listed at the top of the figure.

A

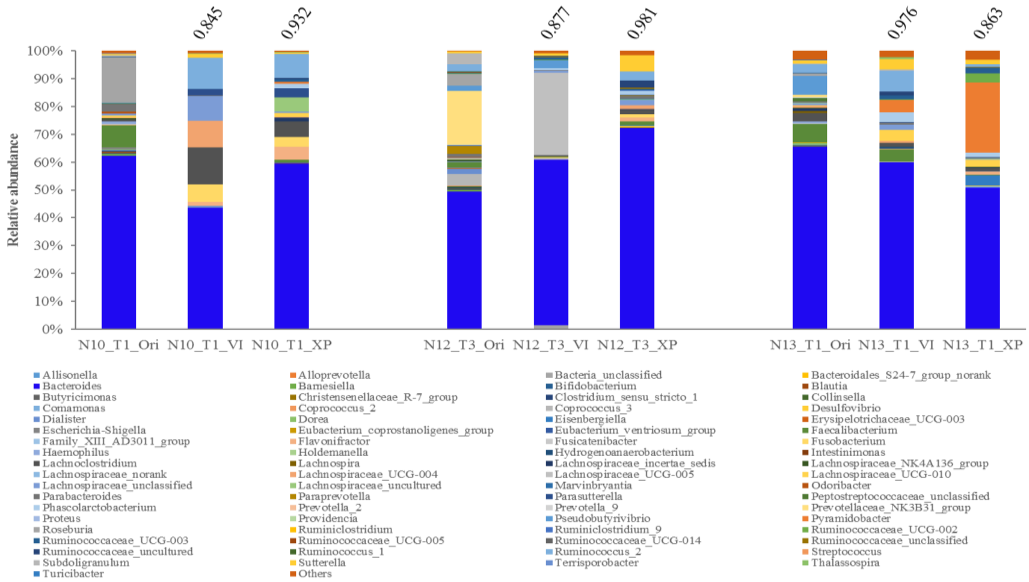

B

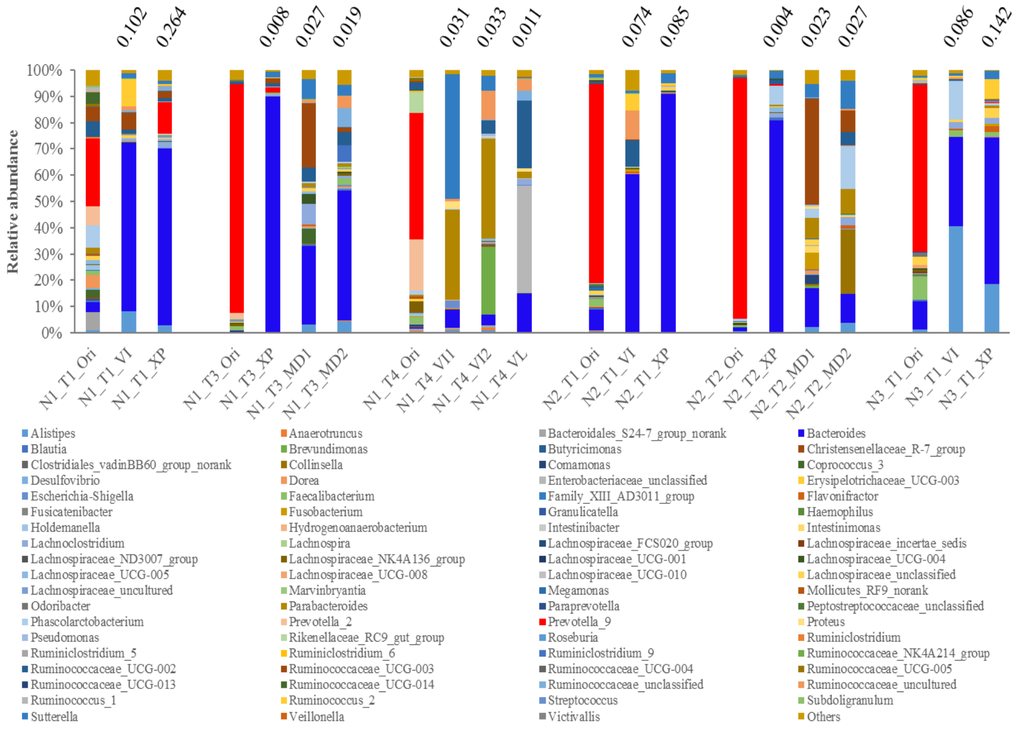
0.102
0.264
0.008
0.027
0.019
0.031
0.033
0.011
0.074
0.085
0.004
0.023
0.027
0.086
0.142
Relative abundance
100%
90%
80%
70%
60%
50%
40%
30%
20%
10%
0%
N1_T1_Ori
N1_T1_VI
N1_T1_XP
N1_T3_Ori
N1_T3_XP
N1_T3_MD1
N1_T3_MD2
N1_T4_Ori
N1_T4_VI1
N1_T4_VI2
N1_T4_VL
N2_T1_Ori
N2_T1_VI
N2_T1_XP
N2_T2_Ori
N2_T2_XP
N2_T2_MD1
N2_T2_MD2
N3_T1_Ori
N3_T1_VI
N3_T1_XP
Alistipes
Anaerotruncus
Bacteroidales_S24-7_group_norank
Bacteroides
Blautia
Brevundimonas
Butyricimonas
Christensenellaceae_R-7_group
Clostridiales_vadinBB60_group_norank
Collinsella
Comamonas
Coprococcus_3
Desulfovibrio
Dorea
Enterobacteriaceae_unclassified
Erysipelotrichaceae_UCG-003
Escherichia-Shigella
Faecalibacterium
Family_XIII_AD3011_group
Flavonifractor
Fusicatenibacter
Fusobacterium
Granulicatella
Haemophilus
Holdemanella
Hydrogenoanaerobacterium
Intestinibacter
Intestinimonas
Lachnoclostridium
Lachnospira
Lachnospiraceae_FCS020_group
Lachnospiraceae_incertae_sedis
Lachnospiraceae_ND3007_group
Lachnospiraceae_NK4A136_group
Lachnospiraceae_UCG-001
Lachnospiraceae_UCG-004
Lachnospiraceae_UCG-005
Lachnospiraceae_UCG-008
Lachnospiraceae_UCG-010
Lachnospiraceae_unclassified
Lachnospiraceae_uncultured
Marvinbryantia
Megamonas
Mollicutes_RF9_norank
Odoribacter
Parabacteroides
Paraprevotella
Peptostreptococcaceae_unclassified
Phascolarctobacterium
Prevotella_2
Prevotella_9
Proteus
Pseudomonas
Rikenellaceae_RC9_gut_group
Roseburia
Ruminiclostridium
Ruminiclostridium_5
Ruminiclostridium_6
Ruminiclostridium_9
Ruminococcaceae_NK4A214_group
Ruminococcaceae_UCG-002
Ruminococcaceae_UCG-003
Ruminococcaceae_UCG-004
Ruminococcaceae_UCG-005
Ruminococcaceae_UCG-013
Ruminococcaceae_UCG-014
Ruminococcaceae_unclassified
Ruminococcaceae_uncultured
Ruminococcus_1
Ruminococcus_2
Streptococcus
Subdoligranulum
Sutterella
Veillonella
Victivallis
Others

C

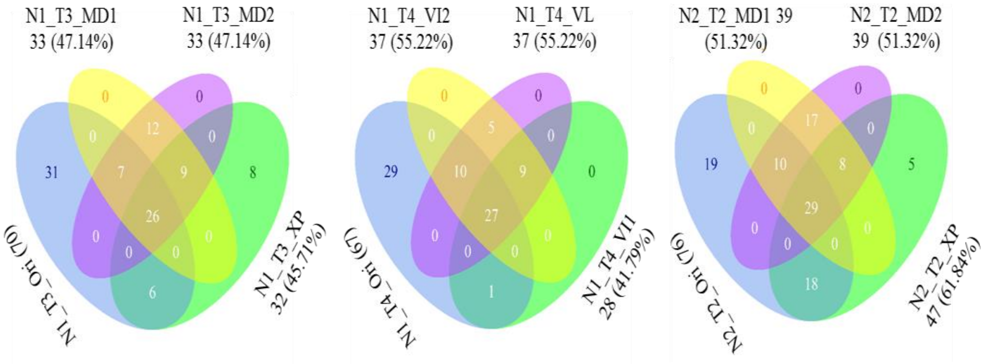
N1_T3_MD1
33 (47.14%)
N1_T3_MD2
33 (47.14%)
N1_T3_Ori (70)
N1_T3_XP
32 (45.71%)
N1_T4_VI2
37 (55.22%)
N1_T4_VL
37 (55.22%)
N1_T4_Ori (67)
N1_T4_VI1
28 (41.79%)
N2_T2_MD1 39
(51.32%)
N2_T2_MD2
39 (51.32%)
N2_T2_Ori (76)
N2_T2_XP
47 (61.84%)

Supplementary Table 1. Basic information regarding the volunteers who participated in this study.

Volunteers	Date of birth	Sex	Sample original	Sample collection time	Sample ID
N1	1978.12	M	Hangzhou	20150109 (T1)	LW20150109
				20150110 (T2)	LW20150110
				20150508 (T3)	LW20150508
				20150730 (T4)	LW20150730
				20160216 (T5)	LW20160216
N2	1990.09	F	Hangzhou	20150202 (T1)	CXX20150202
				20150605 (T2)	CXX20150605
				20151020 (T3)	CXX20151020
N3	1990.09	F	Hangzhou	20150131 (T1)	WYS20150131
				20151020 (T2)	WYS20151020
				20151215 (T3)	WYS20151215
N4	1982.07	M	Hangzhou	20160321 (T1)	YYS20160321
N5	1983.12	F	Hangzhou	20160321 (T1)	HHC20160321
N6	2002.05	M	Beijing	20150407 (T1)	CHN20150407
				20150424 (T2)	CHN20150424
				20150610 (T3)	CHN20150610
N7	1983.09	F	Beijing	20150424 (T1)	WXX20150424
				20150610 (T2)	WXX20150610
N8	1989.12	F	Beijing	20150424 (T1)	WXL20150424
				20150610 (T2)	WXL20150610
N9	1975.12	F	Beijing	20150424 (T1)	LXJ20150424
				20150610 (T2)	LXJ20150610
N10	1991.03	M	Hangzhou	20150113 (T1)	SQS20150113
N11	1992.07	M	Hangzhou	20151020 (T1)	BSF20151020
				20160220 (T2)	BSF20160220
N12	1989.09	M	Hangzhou	20141215 (T1)	FB20141215
				20151020 (T2)	FB20151020
N13	1990.09	M	Hangzhou	20141124 (T1)	TXZ20141124
				20151020 (T2)	TXZ20151020
				20151215 (T3)	TXZ20151215

Supplementary Table 2. Basic statistical results for high-throughput 16S rRNA gene sequencing.

Sample ID	Sequencing platform	Primers	Reads	OUT (0.97)	Ace	Chao	Coverage	Shannon	Simpson
BSF20151020_Ori	Miseq V3-V4	338F_806R	38458	232	275	288	99.89%	3.67	0.05
BSF20160220_Ori	Miseq V3-V4	338F_806R	52090	211	232	228	99.95%	3.54	0.05
CXX20150202_Ori	Miseq V3-V4	338F_806R	25840	186	218	221	99.86%	1.68	0.51
CXX20150202_VI	Miseq V3-V4	338F_806R	24582	79	95	90	99.93%	2.01	0.26
CXX20150202_XP	Miseq V3-V4	338F_806R	21749	80	93	95	99.93%	2.26	0.18
CXX20150605_MD1	Miseq V3-V4	338F_806R	40618	127	153	166	99.94%	2.74	0.18
CXX20150605_MD2	Miseq V3-V4	338F_806R	30353	133	156	160	99.91%	2.81	0.11
CXX20150605_Ori	Miseq V3-V4	338F_806R	47375	173	213	214	99.91%	1.70	0.32
CXX20150605_XP	Miseq V3-V4	338F_806R	39814	132	202	211	99.91%	1.44	0.54
CXX20151020_Ori	Miseq V3-V4	338F_806R	47103	205	228	236	99.93%	2.30	0.21
FB20141215_Ori	Miseq V3-V4	338F_806R	22390	173	256	216	99.82%	3.29	0.07
FB20141215_VI	Miseq V3-V4	338F_806R	31613	101	126	121	99.92%	2.23	0.18
FB20141215_XP	Miseq V3-V4	338F_806R	28072	130	197	172	99.89%	2.47	0.25
FB20151020_Ori	Miseq V3-V4	338F_806R	51570	176	205	205	99.94%	2.39	0.23
LW20150109_Ori	Miseq V3-V4	338F_806R	35751	274	320	349	99.86%	3.63	0.07
LW20150110_Ori	Miseq V3-V4	338F_806R	31081	254	293	294	99.85%	3.60	0.07
LW20150109_VI	Miseq V3-V4	338F_806R	32789	91	100	98	99.96%	1.83	0.36
LW20150109_XP	Miseq V3-V4	338F_806R	32290	137	156	156	99.93%	2.77	0.14
LW20150508_MD1	Miseq V3-V4	338F_806R	22723	118	137	144	99.91%	2.88	0.11
LW20150508_MD2	Miseq V3-V4	338F_806R	32067	126	142	150	99.94%	2.88	0.14
LW20150508_Ori	Miseq V3-V4	338F_806R	28035	172	201	195	99.86%	1.79	0.40

Sample ID	Sequencing platform	Primers	Reads	OUT (0.97)	Ace	Chao	Coverage	Shannon	Simpson
LW20150508_XP	Miseq V3-V4	338F_806R	27028	107	121	124	99.93%	2.04	0.24
LW20160216_Ori	Miseq V3-V4	338F_806R	23392	183	218	238	99.82%	2.51	0.18
SQS20150113_Ori	Miseq V3-V4	338F_806R	32066	162	175	173	99.94%	2.88	0.11
SQS20150113_VI	Miseq V3-V4	338F_806R	37182	85	97	92	99.97%	2.55	0.13
SQS20150113_XP	Miseq V3-V4	338F_806R	28887	100	109	107	99.96%	3.06	0.07
TXZ20141124_Ori	Miseq V3-V4	338F_806R	32964	177	200	200	99.92%	2.81	0.16
TXZ20141124_VI	Miseq V3-V4	338F_806R	40795	108	124	125	99.96%	2.64	0.14
TXZ20141124_XP_5	Miseq V3-V4	338F_806R	38741	120	130	130	99.96%	2.61	0.14
TXZ20151020_Ori	Miseq V3-V4	338F_806R	44441	204	249	269	99.90%	2.69	0.12
TXZ20151215_Ori	Miseq V3-V4	338F_806R	30686	189	204	202	99.92%	2.85	0.11
WYS20150131_Ori	Miseq V3-V4	338F_806R	24398	169	185	185	99.90%	2.88	0.12
WYS20150131_VI	Miseq V3-V4	338F_806R	30371	96	113	134	99.94%	2.29	0.19
WYS20150131_XP	Miseq V3-V4	338F_806R	28243	104	128	129	99.92%	2.66	0.12
WYS20151020_Ori	Miseq V3-V4	338F_806R	48713	197	217	223	99.94%	2.60	0.15
WYS20151215_Ori	Miseq V3-V4	338F_806R	56312	204	216	215	99.96%	3.28	0.08
YYS20160321_Ori	Miseq V3-V4	338F_806R	40123	101	106	110	99.98%	1.93	0.31
HHC20160321_Ori	Miseq V3-V4	338F_806R	50948	90	90	90	100.00%	2.57	0.17
LW20150730_Ori	Miseq V4-V5	515F_907R	32825	115	119	122	99.98%	2.53	0.15
LW20150730_VI1	Miseq V4-V5	515F_907R	34442	60	69	69	99.97%	1.95	0.23
LW20150730_VI2	Miseq V4-V5	515F_907R	32871	87	87	87	100.00%	2.35	0.19
LW20150730_VL	Miseq V4-V5	515F_907R	33310	71	76	82	99.98%	2.09	0.24
CHN20150407_Ori	Miseq V3-V4	338F_806R	26396	219	252	248	99.86%	3.57	0.05
CHN20150424_Ori	Miseq V3-V4	338F_806R	36544	190	209	213	99.93%	3.06	0.10
CHN20150610_Ori	Miseq V3-V4	338F_806R	26449	201	236	230	99.86%	3.16	0.12
WXX20150424_Ori	Miseq V3-V4	338F_806R	25140	176	192	191	99.91%	3.49	0.06

Sample ID	Sequencing platform	Primers	Reads	OUT (0.97)	Ace	Chao	Coverage	Shannon	Simpson
WXX20150610_Ori	Miseq V3-V4	338F_806R	46264	169	187	187	99.95%	2.79	0.17
WXL20150424_Ori	Miseq V3-V4	338F_806R	29597	177	191	191	99.93%	3.51	0.05
WXL20150610_Ori	Miseq V3-V4	338F_806R	48006	189	195	194	99.98%	3.47	0.06
LXJ20150424_Ori	Miseq V3-V4	338F_806R	28801	304	324	330	99.88%	4.22	0.03
LXJ20150610_Ori	Miseq V3-V4	338F_806R	45351	254	319	311	99.87%	2.12	0.23
WXX20150610_Ori_ HiSeq	HiSeq PE250 V4	515F_806R	49517	379	386	387	99.96%	3.60	0.10
WXL20150610_Ori_ HiSeq	HiSeq PE250 V4	515F_806R	47456	348	375	383	99.91%	3.59	0.06
LXJ20150610_Ori_ HiSeq	HiSeq PE250 V4	515F_806R	50503	350	388	403	99.89%	1.90	0.49
